# Unveiling the antifungal arsenal: proteomic profiling of tomato exosomes

**DOI:** 10.1007/s00425-026-05050-7

**Published:** 2026-06-25

**Authors:** Dina Salem, Ahmed Helmy, Mira Mohamed, Nour Moustafa, Marina Wafy, Engy Gergis, Hala F. Eissa

**Affiliations:** 1 Department of Agriculture Biotechnology, College of Biotechnology, Misr University for Science and Technology, P.O. Box 77, Giza, 12566 Egypt; 2 Department of Medical Biotechnology, College of Biotechnology, Misr University for Science and Technology, P.O. Box 77, Giza, 12566 Egypt

**Keywords:** Antifungal activity, *Botrytis cinerea*, Dynamic light scattering, Exosome, *Fusarium oxysporum*, *Fusarium solani*, LC–MS/MS proteomics, *Solanum lycopersicum*, Spore density, Spore germination, Stress-responsive proteins, Transmission electron microscopy

## Abstract

**Main conclusion:**

**Tomato-derived extracellular vesicles selectively package antifungal defense proteins and significantly suppress fungal spore growth and germination, supporting their role as coordinated plant immune delivery systems.**

**Abstract:**

Extracellular vesicles (EVs) are significant facilitators of plant–pathogen communication. However, their role as organized antifungal protein delivery systems is not fully understood. This study investigated whether tomato (*Solanum lycopersicum*) EVs have a unique set of antifungal proteins that helps prevent the growth of phytopathogenic fungi. EVs were extracted from mature tomato fruits and characterized using transmission electron microscopy and dynamic light scattering. They were then analyzed through LC–MS/MS-based proteomic profiling. We identified 133 high-confidence proteins in total; several are involved in defense mechanisms, including pathogenesis-related proteins, defensins, endochitinases, glucanases, osmotin-like proteins, and lipid transfer proteins. Proteins involved in quality control, vesicle trafficking, and metabolic regulation were also enriched. This suggests that EV cargo may participate in stress-responsive and defense-related processes. These functional categories are commonly associated with plant immune responses. Biological assays showed that the density and germination of *Fusarium oxysporum, Fusarium solani,* and *Botrytis cinerea* spores were reduced in a dose-dependent manner. These results bolster the hypothesis that tomato-derived EVs are enriched with antifungal-associated proteins and may serve as coordinated delivery vehicles in plant defense responses. Independent proteomic analysis of EV cargo may contribute to antifungal responses that are not apparent from total secretome analysis alone. The present research improves our understanding of how vesicles help plants fight disease and indicates how plant EVs could be used in long-term disease control strategies. Overall, these findings highlight the potential of plant-derived EVs as innovative, biologically driven tools for enhancing crop protection and developing sustainable antifungal strategies in agriculture**.**

**Supplementary Information:**

The online version contains supplementary material available at 10.1007/s00425-026-05050-7.

## Introduction

Fungal phytopathogens like *Fusarium oxysporum*, *Fusarium solani*, and *Botrytis cinerea* cause severe tomato losses. Plants use layered defenses: antimicrobial proteins, cell wall fortification, oxidative bursts, and RNA interference (Engelsdorf et al. 2018). The targeting and organization of these defenses remain unclear.

Extracellular vesicles (EVs), including plant-derived exosomes, are membrane-bound nanostructures released into the apoplast and extracellular space. Their diameters vary by EV type, generally ranging from 50 nm to 1 µm. EVs participate in cell signaling and multiple physiological and pathological mechanisms (Yáñez-Mó et al. 2015), including signal transduction (Li et al. 2021; Chimal-Vega et al. 2025), cell cycle regulation (Zhang et al. 2021), immunological response (Hussain et al. 2022; Gangadaran et al. 2023), neurological disorders (Alzahrani et al. 2024), inflammation (Gao et al. 2022a; Zhao et al. 2023; Saleem et al. 2024), and cancer (Dai et al. 2020; Stanly et al. 2020). Recent studies suggest that plant EVs transport proteins, lipids, and small RNAs during plant–microbe interactions. For example, EV-mediated transfer of small RNAs from plants to fungal pathogens can suppress virulence genes, and defense-related proteins have been detected in EV preparations from several plant species (Rybak And Robatzek 2019; He et al. 2021; Garaeva et al. 2021). Proteomic analysis of sunflower seedling EVs revealed proteins involved in defense, including pathogenesis-related (PR) proteins and disease resistance factors such as PMR5 and the antifungal protein Gnk2 (Regente et al. 2017).

Plant-derived extracellular vesicles (P-EVs) are less well studied than mammalian extracellular vesicles, particularly with respect to their proteomic composition and functional roles in plant–pathogen interactions (Huang et al. 2025; Salem et al. 2025). Electron microscopy shows plant EVs play key roles in local immune responses. Multivesicular bodies (MVBs) increase and move to infection sites during fungal and bacterial attack. Like animal exosomes, MVBs merge with the membrane, releasing intraluminal vesicles into the apoplast (Manocha And Shaw 1964; An et al. 2006; Wang et al. 2014).

EV lipids are viewed as active communication molecules, shaped by their local environment. They should be recognized as a key part of the signaling mechanisms enabling cellular interactions. The lipidome of EVs can change in response to internal and external biological stimuli (Harizi et al. 2008; Zhang et al. 2025).

Even with these improvements, a significant knowledge gap remains. Many antifungal proteins, such as PR proteins, defensins, glucanases, chitinases, and lipid transfer proteins, have been identified in total secretome or apoplastic fluids (Boudart et al. 2005). However, it is unclear if these proteins are specifically encapsulated in EVs. It is also unknown whether EV cargo differs quantitatively or qualitatively from the overall secretome, or whether vesicle-associated proteins directly enhance antifungal activity.

If researchers do not examine EV proteomes separately, vesicle-enriched proteins, coordinated protein complexes, and compartmentalized delivery mechanisms might be overlooked. Analyzing total extracellular proteins cannot distinguish between freely secreted proteins and those selectively enclosed in vesicles (Bifolco et al. 2026). Therefore, researchers must conduct proteomic profiling of purified extracellular vesicles. This distinction helps determine if vesicles form an organized antifungal delivery system or are simply passive debris.

Tomato (*Solanum lycopersicum*) is a crop that is highly susceptible to fungal pathogens (Savary et al. 2019; Falade And Alabi 2025). While EVs have been detected in the roots and leaves of some species, comprehensive proteomic analysis of tomato fruit EVs for antifungal activity remains limited.

The principal research inquiry of this study is: Do EVs from tomatoes have a unique set of antifungal proteins that help stop phytopathogenic fungi from growing? To answer this question, we (1) separated and characterized extracellular vesicles from tomato fruit, (2) performed proteomic profiling of purified vesicles using LC–MS/MS, (3) carried out functional enrichment analysis of EV-associated proteins, (4) evaluated the antifungal activity of tomato-derived EVs against *Fusarium oxysporum*, *Fusarium solani*, and *Botrytis cinerea*, and (5) determined whether the proteomic cargo could elucidate potential mechanisms underlying the observed antifungal activity. This study investigates whether tomato EVs represent a uniquely coordinated antifungal protein delivery system, distinct from the general secretome, through an integrated approach combining EV-targeted proteomics and biological assays.

## Materials and methods

### Exosome isolation

Ripe red tomatoes (*Solanum lycopersicum*, cv. Super Strain B) were purchased from a local market in Giza, Egypt. The fruits were fully ripe at the red-ripe stage, the usual postharvest stage for consumption. This study focuses on fruit vesicles. The findings clarify EV cargo in mature tomato tissues and offer a benchmark for further studies into vesicle dynamics in developing or infected plants.

The fruit juice was centrifuged first at 3000 g for 10 min, then at 10,000 g for 20 min. The supernatant was filtered using a 0.22-μm pore filter to isolate tomato exosomes. The filtered juice was centrifuged at 100,000 g at 4 °C for 1 h to pellet the exosomes. To purify the exosomes, the pellet was resuspended in ultrapure water produced by a Milli-Q water purification system (MilliporeSigma, Burlington, MA, USA), with a resistivity of 18.2 MΩ cm at 25 °C and total organic carbon (TOC) < 5 ppb. It was then centrifuged again at 100,000 g for 2 h. The particles were finally resuspended in Milli-Q water.

### Exosome characterization

#### Dynamic light scattering (DLS)

The size distribution of the isolated exosomes was measured by DLS with a Zetasizer Nano ZS Zen3600 (Malvern Panalytical Ltd., Malvern, UK). Isolated exosomes were diluted 1000-fold with Milli-Q water. The diluted sample was placed in a 12 mm square polystyrene cuvette (DTS0012, Malvern). Measurements were done in triplicate at 37 °C.

#### Transmission electron microscopy (TEM)

Isolated EVs were characterized by TEM using a JEOL JEM-2100F microscope (JEOL Ltd., Tokyo, Japan) at 200 kV. Samples were prepared by placing a drop of a dilute suspension (0.05 mg/ml in ethanol) onto carbon-coated copper grids and allowing them to dry at room temperature. TEM gave direct images of the nanomaterials’ size, shape, and internal structure.

### LC–MS/MS proteomic analysis

#### Protein extraction

Protein extraction from tomato fruit exosomes was carried out by the following steps: Initially, 100 μl of 8 M urea (500 mM Tris pH 8.5) was mixed with 50 μl of tomato exosomes.

The mixture was centrifuged at 8000 g for thirty min, after which the supernatant was utilized for the remaining steps. The concentration of total proteins was measured using the bicinchoninic acid assay kit (Pierce™ BCA Protein Assay Kit, Thermo Fisher Scientific, Waltham, MA, USA), according to the manufacturer’s instructions.

#### Protein digestion and peptide preparation

Proteomic sample preparation and LC–MS/MS analysis were performed at the Proteomics and Metabolomics Research Program at the Children’s Cancer Hospital (Egypt).

Protein samples were adjusted to a final volume of 30 μl prior to digestion. Reduction was carried out by adding dithiothreitol (DTT) to a final concentration of approximately 12.5 mM; the mixture was vortexed and was permitted to stand at 37 °C for 45 min. Subsequently, 2 μl of 1 M iodoacetamide was added at approximately 62.5 mM and the mixture was left at room temperature in the dark for 45 min. Finally, 102 μl of 100 mM Tris (pH 8.5) and 6 μl of trypsin (Promega, Madison, WI, USA) were added at an approximate enzyme-to-protein ratio of 1:50 (w/w) and thereafter the mixture was incubated for 12 h at 37 °C in a shaker incubator (900 rpm). The reaction was terminated by acidification using formic acid to a final concentration of approximately 2–3% (v/v) (pH 2–3) (Magdeldin et al. 2014; Saadeldin et al. 2020). Peptides were purified using MonoSpin reversed-phase C18 columns (GL Sciences, Inc., Tokyo, Japan; product no. 5010–21701) according to the manufacturer’s protocol. Eluted peptides were dried and reconstituted in 0.2% formic acid prior to LC–MS/MS analysis.

#### Liquid chromatography

Peptide separation was performed using a nanoLC system consisting of an Eksigent nanoLC 400 autosampler coupled to an Ekspert nanoLC 425 pump (SCIEX, Marlborough, MA, USA). Separation was carried out on a ChromXP C18CL analytical column (3 μm, 150 × 0.3 mm) with a ChromXP C18CL trap column (5 μm, 10 × 0.5 mm). A total of 1 μg peptide was injected, and the flow rate was maintained at 5 μl/min. Mobile phase A consisted of LC–MS-grade water containing 0.1% (v/v) formic acid, whereas mobile phase B consisted of acetonitrile containing 0.1% (v/v) formic acid. Peptides were separated using a 55-min gradient as follows: 3% B at 0 min, increased to 30% B over 38 min, increased to 40% B at 43 min, increased to 80% B at 45 min and maintained until 48 min, then returned to 3% B at 49 min and re-equilibrated until 55 min.

Mobile phase A consisted of LC–MS-grade water containing 0.1% (v/v) formic acid, while mobile phase B consisted of acetonitrile containing 0.1% (v/v) formic acid. Peptides were separated using a 55-min gradient at a flow rate of 5 μl/min.

The gradient profile was 0 min-3%B, 38 min-30%B, 43 min-40%B, 45–48 min-80%B, 49–57 min-3%B, and the total run time 55 min.

#### Mass spectrometry

Mass spectrometry was performed using a TripleTOF™ 5600 + mass spectrometer (SCIEX) operating in positive-ion mode. Data were acquired using an Information-Dependent Acquisition (IDA) method (DDA-type), with an MS1 scan range of 400–1250 m/z, an MS2 scan range of 170–1500 m/z, selection of the top 40 precursor ions per cycle, a cycle time of 1.5 s, and an ion selection threshold of 150 cps.

### Data processing and protein identification

Raw data acquisition was performed using Analyst TF 1.7.1 software (SCIEX), and protein identification was conducted using ProteinPilot™ Software v5.0.1 (SCIEX) with the Paragon algorithm. Spectra were searched against the UniProt *Solanum lycopersicum* database (Swiss-Prot and TrEMBL; 52,323 entries). Search parameters included trypsin as the digestion enzyme, carbamidomethylation (C) as a fixed modification, and biological modifications as variable modifications. The search effort was set to thorough, mass type was monoisotopic, false discovery rate (FDR) analysis was enabled using a target-decoy strategy, and bias correction was enabled. Protein identification confidence was assessed using ProteinPilot scoring.

### Protein identification criteria and filtering

Protein identification was based on high-confidence peptide matches, removal of reverse decoy sequences, and filtering according to peptide evidence. Proteins lacking peptide support, defined as Peptides (95%) = 0, were excluded from the final dataset.

### Supplementary data and scoring interpretation

The supplementary dataset was expanded to include peptide-level information, including the number of peptide spectral matches, peptide sequences, charge states, mass-to-charge ratios, protonated molecular ion masses, mass error (ΔM), post-translational modifications, match scores, and confidence values. ProteinPilot scoring parameters were interpreted as follows: the Unused score reflects protein identification confidence based on unique peptides, whereas the Total score includes both unique and shared peptide evidence.

The gene enrichment of three ontologies—biological processes, cellular components, and molecular functions—was evaluated using the right-tailed Fisher’s exact test. Gene Ontology (GO) analysis was performed using ShinyGO 0.81 (South Dakota State University, Brookings, SD, USA), which provides an application programming interface to Kyoto Encyclopedia of Genes and Genomes (KEGG) and STRING. A ShinyGO program, based on the R/Bioconductor software, employs an extensive pathway database. The difference was interpreted as significant when *p* < 0.05.

### Assessment of antifungal activity

The activity of tomato exosomes against phytopathogens was first assessed using the disk diffusion method. Following the acquisition of the fungal strains *Fusarium oxysporum* f.sp. *radicis lycopersici*, *Fusarium solani*, and *Botrytis cinerea* from the Microbial Resource Center (MIRCEN) at the Faculty of Agriculture, Ain Shams University, Cairo, Egypt, the culture was maintained for 10 days at 25 °C.

After introducing 15 ml of sterile water and disrupting the mycelia, the spore suspension was generated from the culture by filtering the solution through cheesecloth. The spore count was confirmed using a hemocytometer. One hundred microliters of tomato exosome suspension were applied to each disk. After refrigeration for 1 h, the plates were incubated at 37 °C for 24 h. Then, the inhibition zones (ZI) were measured in millimeters.

### Effect of tomato exosomes on the density of spores

Spores of *F. solani, F. oxysporum,* and *B. cinerea* were obtained, and spore concentration was calibrated to 10^6^/ml using the aforementioned technique. Ten ml of PDA were dispensed into test tubes containing three concentrations of tomato exosomes (100, 200, and 400 µg/ml). Subsequently, 100 µl (10^6^ spores/ml) of spore suspension was combined with two components. The cultures were maintained at 28 ± 2 °C (‘room temperature’) in darkness for 120 h.

A hemocytometer was used to count spores. The inhibitory activity on spore production was assessed using the following formula:

$$\mathrm{I}\mathrm{S}=\left(\frac{\mathrm{d}\mathrm{c}-\mathrm{d}\mathrm{t}}{\mathrm{d}\mathrm{c}}\right) \times 100$$

where IS represents the inhibition of spore development, dc denotes the spore density of the control (distilled water only), and dt indicates the spore density following treatment with ELNs (Abbas et al. 2022)**.**

### Effect of tomato exosomes on germination of spores

Following spore capture and adjustment of their concentration to 10^6^/ml, Eppendorf tubes were filled with PDB medium containing three concentrations of tomato exosomes (100, 200, and 400 µg/ml). The resultant combination was mixed with a spore suspension at a concentration of 10^6^spores/ml. Three Eppendorf tubes were evaluated for each concentration. The mixture was incubated for 12 h in the dark at 28 ± 2 °C. The number of germinated spores was quantified by a hemocytometer (Abbas et al. 2022).

### Statistical analysis

The results were presented as means ± standard error of the mean (SE) and analyzed using one-way analysis of variance (ANOVA) to assess differences among group means. Results were interpreted as significant when *P* < 0.05.

## Results

### TEM results

**Fig. 1:**
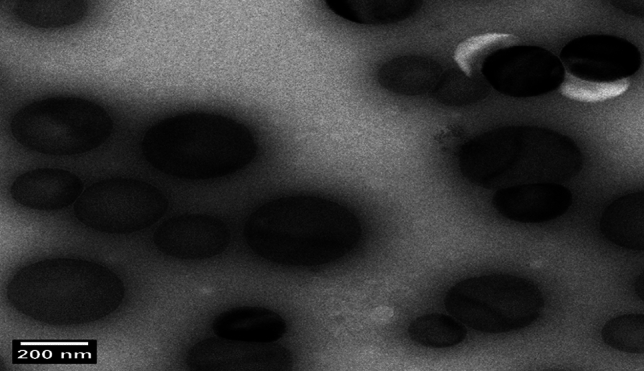
TEM image of the *Solanum lycopersicum* EVs, with diameters ranging from 95 to 231 nm

**Fig. 2:**
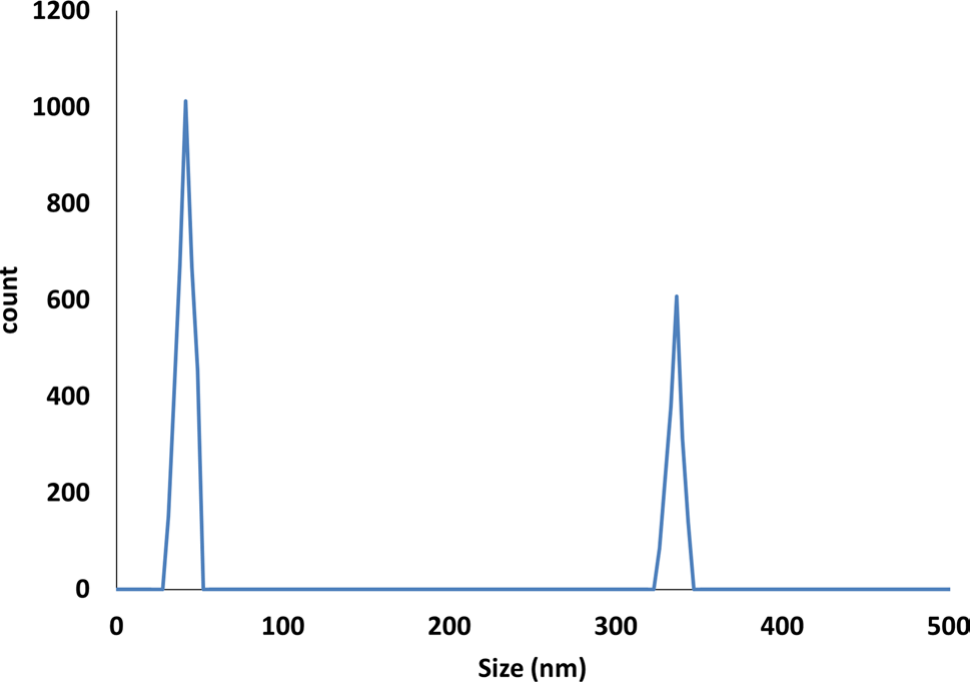
Dynamic light scattering (DLS) of *Solanum lycopersicum* EVs. Two populations exist: the first with sizes between 30 and 52.12 nm, the second ranges from 322.78 to 347.07 nm

Based on TEM data, the diameter of EVs varied from 95 to 231 nm, with an average of 172.2862 nm (Fig. 1). Moreover, DLS findings revealed the existence of two populations: the first has sizes between 30 and 52.12 nm, whereas the second ranges from 322.78 to 347.07 nm as illustrated in Fig. 2.

Although TEM revealed vesicles ranging from 95 to 231 nm, DLS analysis showed two size populations (30–52 nm and 322–347 nm). The larger population detected by DLS may be due to vesicle aggregation in suspension or to microvesicle-like particles. Because DLS measurements are intensity-weighted and sensitive to larger particles, minor aggregation can result in an apparent second size population not clearly resolved by TEM imaging.

### Bioinformatic analysis of identified exosomal proteins

The proteome analysis proved the existence of commonly identified EVs proteins like: carbohydrate metabolism proteins (glyceraldehyde-3-phosphate dehydrogenase, malate dehydrogenase, pyruvate, phosphate dikinase), proteolytic proteins (cysteine proteinase 3, peptidase, lipid transfer proteins), protein synthesis (elongation factors, ribosomal proteins), cytoskeletal (tubulin, actin), and heat shock response proteins (Hsp80, Hsp70, Hsp90). Interestingly, vesicular transport proteins, including annexin and tiny GTPases, were also found. It is worth noting that many of the identified proteins are defense-related as illustrated in Table 1.

**Table 1:** Defense-related proteins of tomato-derived exosomes

Number	Name of protein	Acc. No
1	Low-temperature-induced cysteine proteinase-like	A0A3Q7F355
2	Pathogenesis-related leaf protein 6	P04284
3	Glucan endo-1,3-beta-glucosidase B	Q01413
4	Xyloglucan-specific fungal endoglucanase inhibitor protein	Q8GT67
5	14–3-3 containing protein	A0A3Q7IR67
6	LysM domain-containing protein	A0A3Q7FF75
7	Suberization-associated anionic peroxidase 1	P15003
8	Bet v I/Major latex protein domain-containing protein	A0A3Q7FZ65
9	Abscisic stress-ripening protein 1	Q08655
10	Annexin	O81536
11	14–3-3 protein 10	P93207
12	Manganese/iron superoxide dismutase	A0A3Q7GS80
13	Malate dehydrogenase	A0A3Q7HGQ2
14	Heat shock cognate protein 80	P36181
15	Osmotin-like protein TPM-1	Q01591
16	Osmotin-like protein PR-5x	Q8LPU1
17	Pathogenesis-related leaf protein 4	Q04108
18	Basic 30 kDa endochitinase	Q05538
19	Heat shock cognate 70 kDa protein 2	P27322
20	Heat shock protein 70 isoform 2	H1ZXA8
21	Heat shock protein 70 isoform 1	H1ZXA7
22	Heat shock protein 70 isoform 3	H1ZXA9
23	Uncharacterized protein	A0A3Q7FX57
24	Uncharacterized protein	A0A3Q7FS03
25	Heat shock cognate 70 kDa protein 1	P24629
26	14–3-3 protein 1	P93206
27	Leucine-rich repeat-containing	A0A3Q7JMX3
28	N-terminal plant-type domain-containing protein	A0A3Q7JMX3
29	Cell division cycle protein 48 homolog	A0A3Q7J082
30	Molecular chaperone Hsp90-1	Q6UJX4
31	Molecular chaperone Hsp90-2	Q53Z32
32	Histidine kinase/HSP90-like ATPase domain-containing protein	A0A3Q7HLX5
33	Glyoxalase	A0A3Q7JRP8
34	Temperature-induced lipocalin	Q38JD4
35	Catalase	A0A3Q7JD73
36	Defensin protein	B1N678
37	Knottin scorpion toxin-like domain-containing protein	A0A3Q7H3Y0
38	PLAT domain-containing protein	A0A3Q7G505
39	lipid transfer protein	A0A3Q7HSL7
40	Ethylene-responsive proteinase inhibitor 1	P20076
41	Lipoxygenase	A0A3Q7ENA3
42	Pathogenesis-related protein PR1a (P4)	Q546T9
43	Late embryogenesis (Lea)-like protein	Q40159

An enrichment analysis was performed for gene ontologies and pathways. This signifies that the phrase is more prevalent in the protein set within the network than in the background. We classified the 133 proteins into functional categories (GO biological process, GO cellular component, and GO molecular function) with the ShinyGO v0.81 database. Enrichment analysis accessed at (http://bioinformatics.sdstate.edu/go/). The FDR was calculated using the nominal *P*-value (≤ 0.05). The background gene list used for enrichment analysis comprised the complete *Solanum lycopersicum* proteome (52,323 proteins) retrieved from the UniProt database, serving as the reference set for GO and KEGG comparisons. FDRs decrease across extensive pathways due to increased statistical power. This measure signifies the extent to which particular genes are disproportionately represented within a pathway. The full list of the identified proteins has been provided in Suppl. File S1.

### GO analysis of biological functions

**Fig. 3:**
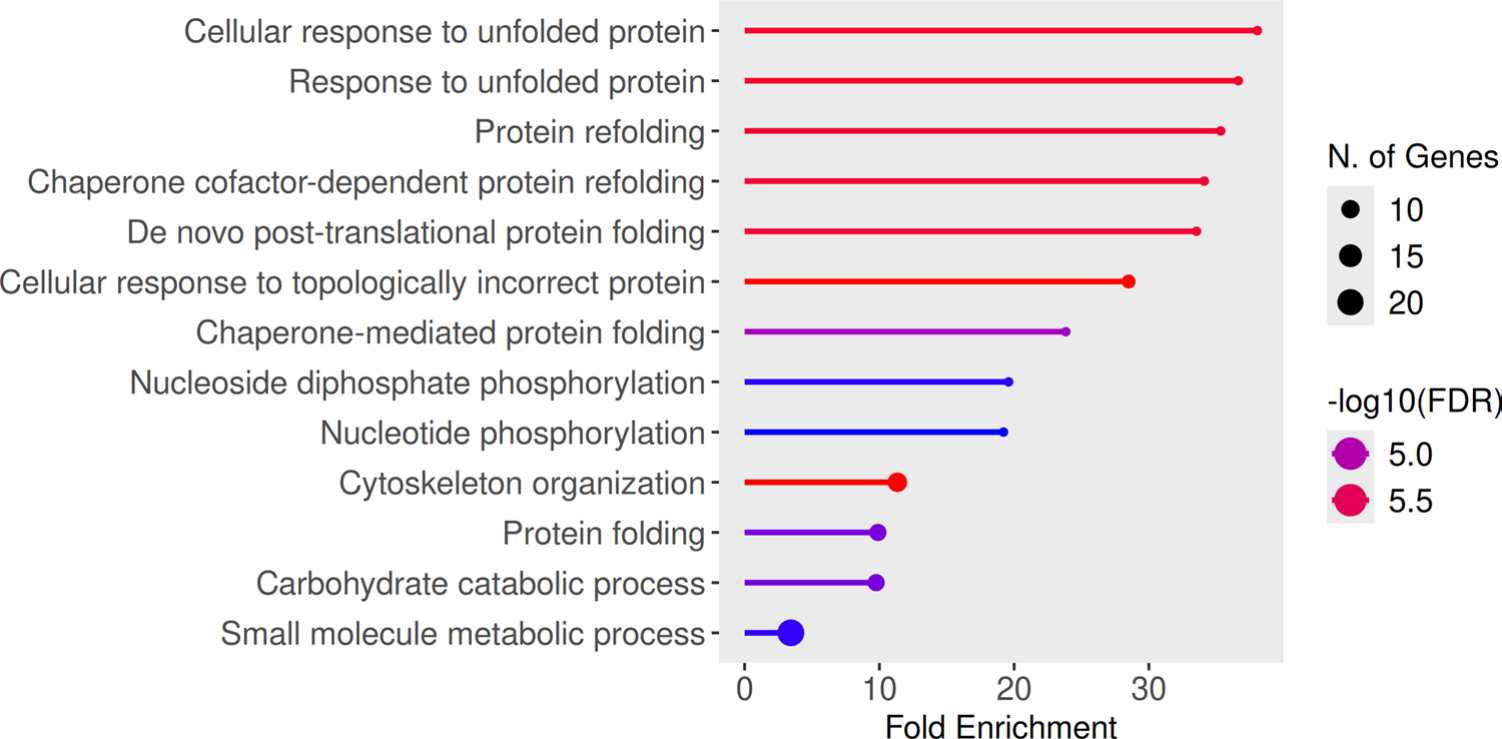
GO analysis of biological processes of *Solanum lycopersicum EVs* identified proteins

The analysis of biological processes (Fig. 3) revealed that the identified proteins are predominantly involved in protein folding and quality control mechanisms, including cellular response to unfolded proteins, protein refolding, and chaperone-mediated folding pathways. Additionally, enrichment was observed in processes related to nucleotide phosphorylation, cytoskeleton organization, and carbohydrate catabolism, as well as broader small-molecule metabolic processes. These findings indicate that EV proteins are strongly associated with protein homeostasis, stress response mechanisms, and cellular structural organization, highlighting their potential role in maintaining cellular stability under stress conditions and supporting metabolic regulation.

### GO analysis of molecular functions

**Fig. 4:**
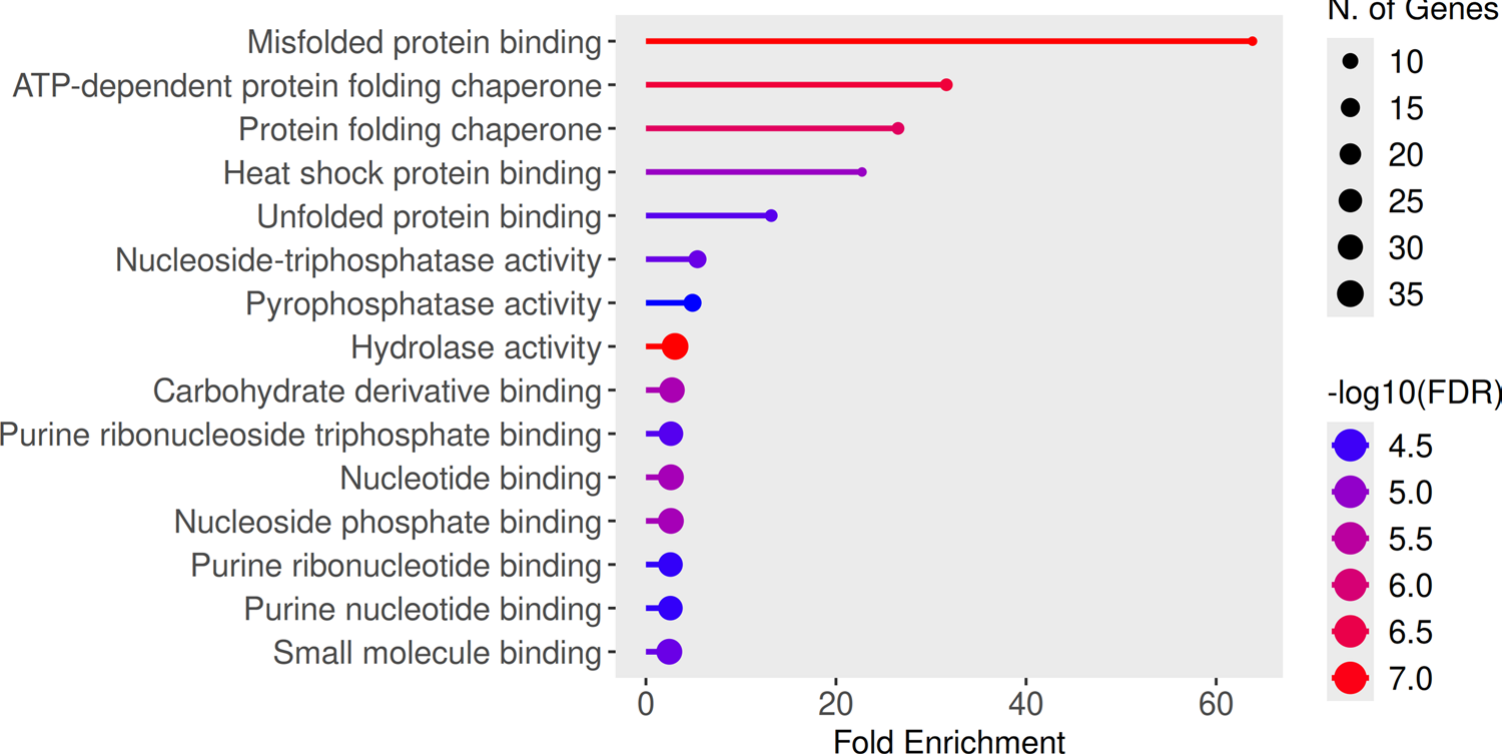
GO analysis of molecular functions of *Solanum lycopersicum* EVs identified proteins

The analysis of molecular functions (Fig. 4) revealed significant enrichment of proteins involved in misfolded protein binding, ATP-dependent chaperone activity, protein folding chaperone functions, and heat shock protein binding, highlighting a strong association with protein quality control mechanisms. Additionally, enrichment was observed in nucleotide and nucleoside phosphate binding, hydrolase and pyrophosphatase activities, and carbohydrate derivative binding, indicating roles in enzymatic activity and molecular interactions. These results suggest that EV proteins primarily maintain protein homeostasis through chaperone-mediated folding and stabilization, while also participating in energy-dependent processes and metabolic regulation. Collectively, this functional profile supports a role for EVs in cellular stress response, protein quality control, and molecular binding interactions essential for cellular function.

### GO analysis of cellular and subcellular components

**Fig. 5:**
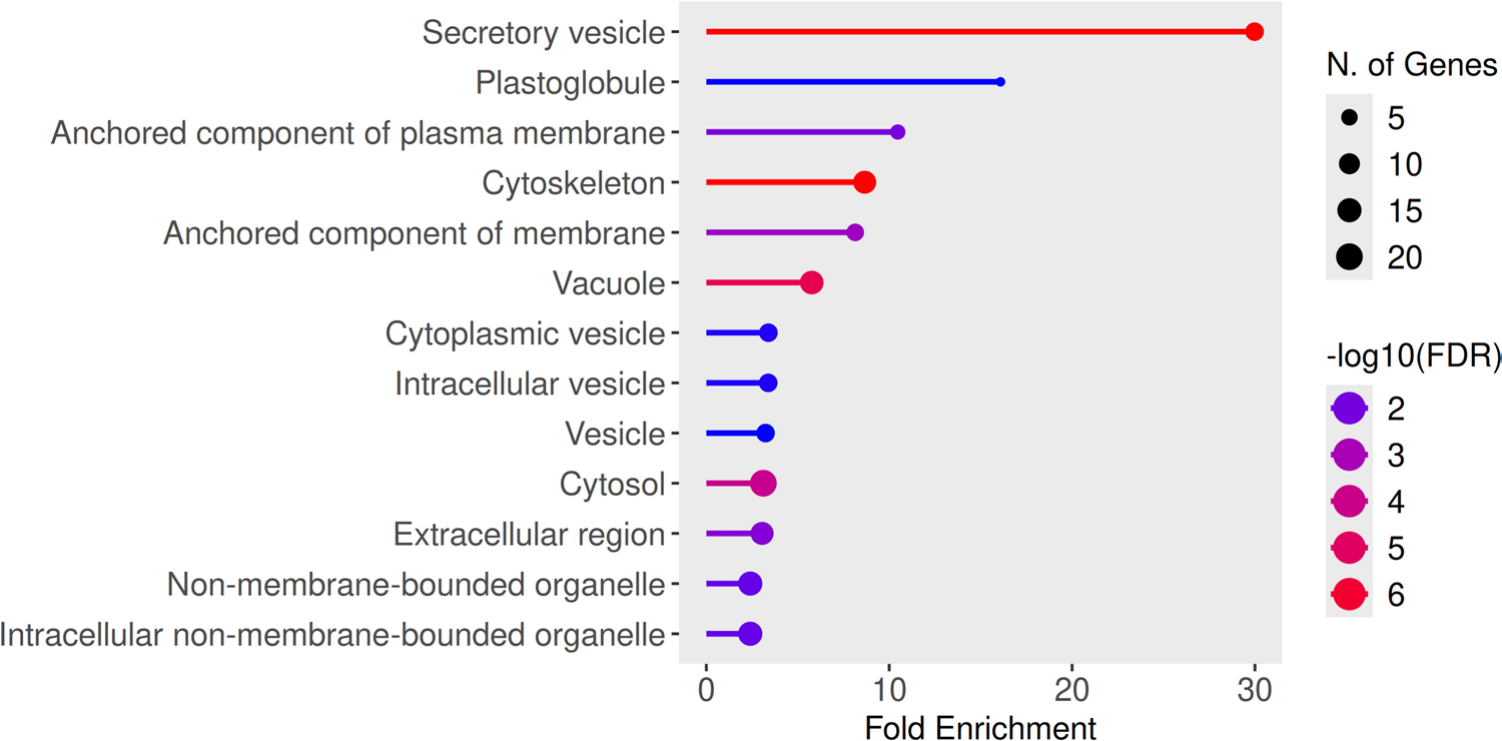
GO Analysis of cellular components of *Solanum lycopersicum* EVs identified proteins

The analysis of cellular components revealed that the identified proteins are predominantly localized to secretory vesicles, plastoglobules, membrane-associated compartments (including anchored components of the plasma membrane), the cytoskeleton, and the vacuole, as well as to cytoplasmic and intracellular vesicles (Fig. 5). Additional enrichment was observed in cytosolic and extracellular regions, along with non-membrane-bounded organelles. These results indicate that EV-associated proteins are mainly distributed across vesicle-mediated transport systems, membrane structures, and intracellular trafficking compartments, highlighting their involvement in secretion, cellular organization, and intercellular communication. The enrichment of cytoskeletal and membrane-associated components further supports a role for these proteins in vesicle formation, transport, and structural dynamics within plant cells.

**Fig. 6:**
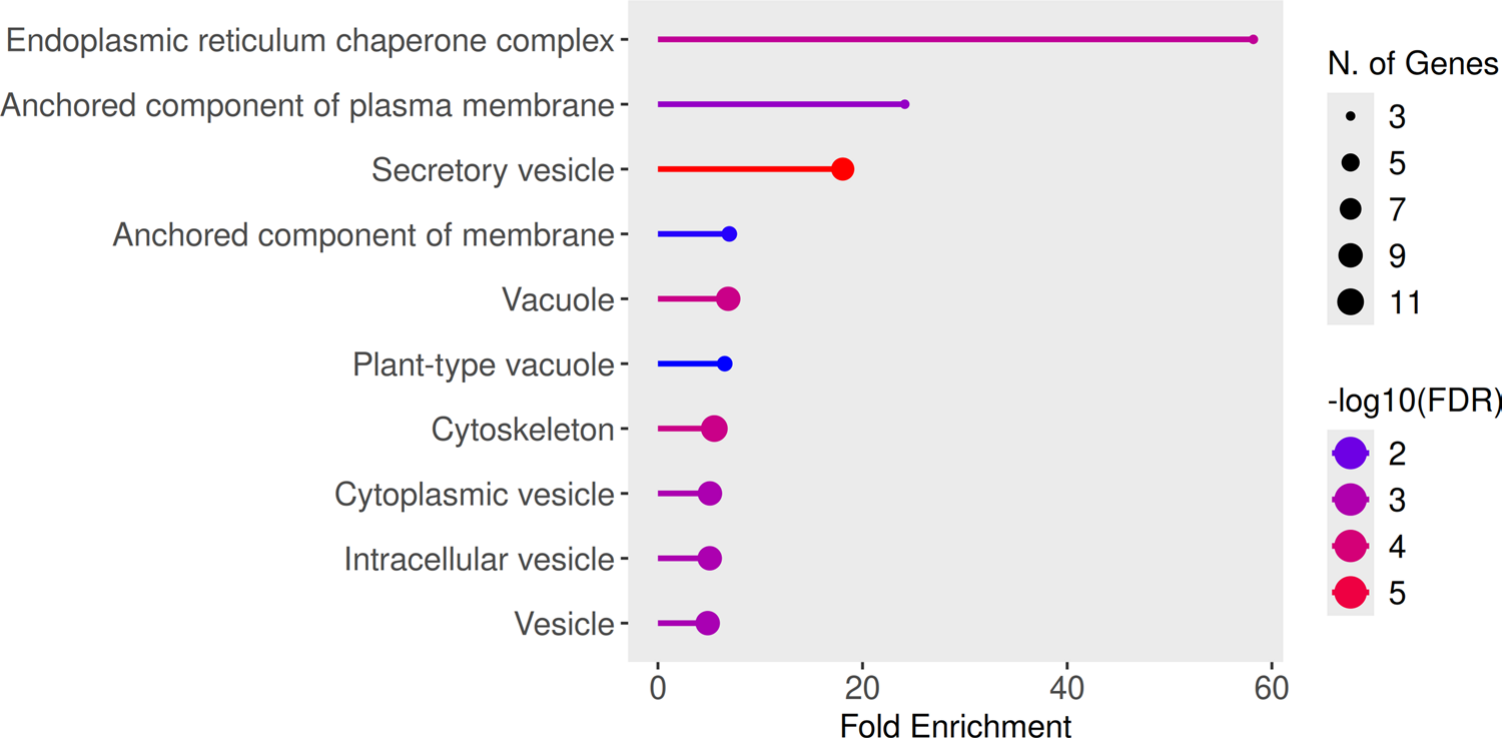
GO analysis of subcellular identified proteins of *Solanum lycopersicum* EVs

The examination of cellular constituents indicated that the detected proteins are markedly abundant in endoplasmic reticulum chaperone complexes, secretory vesicles, and membrane-associated elements, including plasma membrane anchoring components. Enhanced enrichment was noted in vacuolar compartments (including plant-type vacuoles), cytoskeletal structures, and diverse vesicle-related compartments such as cytoplasmic, intracellular, and general vesicles (Fig. 6). The findings demonstrate that proteins associated with EVs are predominantly located in environments for protein folding and processing, vesicle-mediated transport systems, and membrane structures, underscoring their roles in protein quality control, intracellular trafficking, and secretion pathways. The enhancement of cytoskeletal and membrane-associated elements further substantiates their function in vesicle formation, transport, and structural organization inside plant cells.

### KEGG pathway analysis

**Fig. 7:**
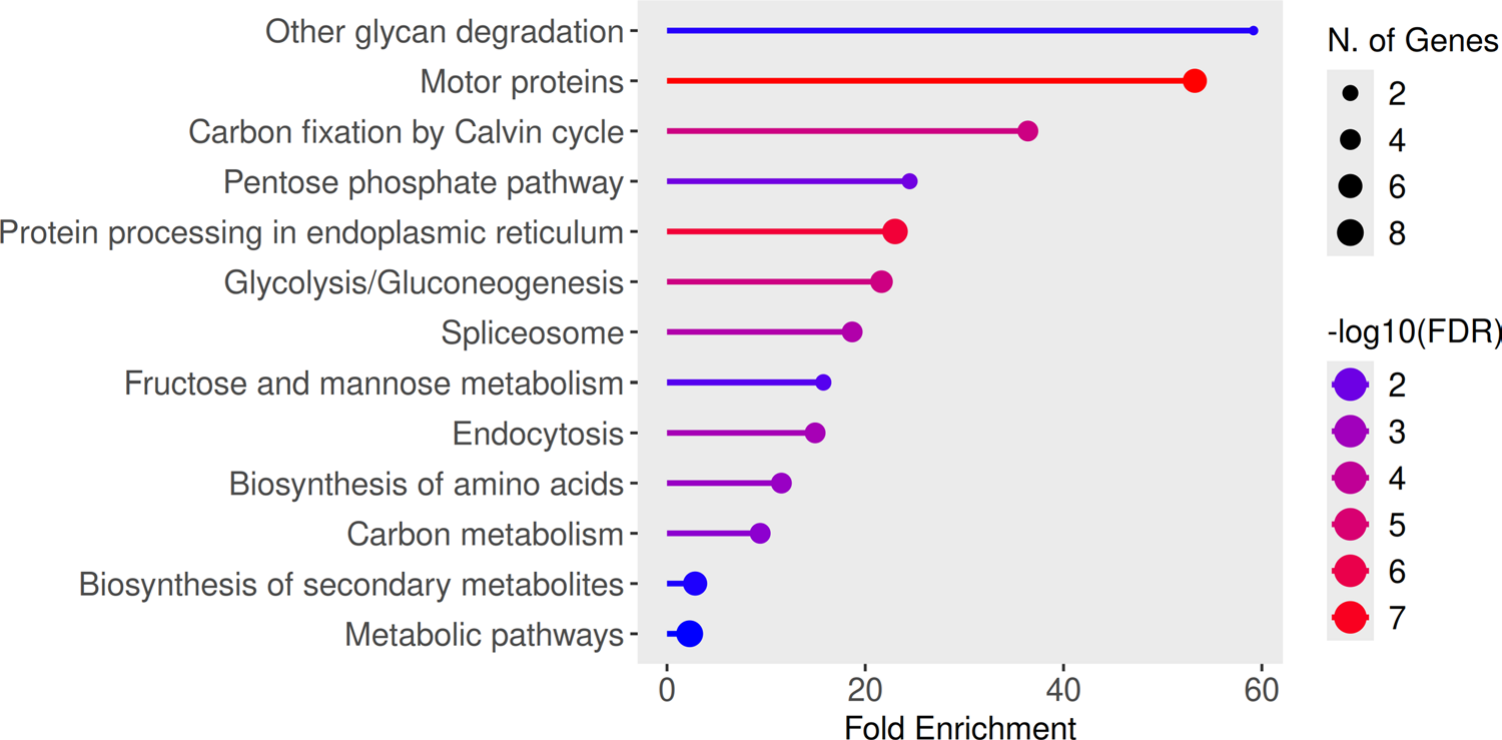
Enriched KEGG in *Solanum lycopersicum* EVs

Figure 7 illustrates the KEGG pathway enrichment analysis of EVs derived from *Solanum lycopersicum*, highlighting both fold enrichment and statistical significance (− log10 FDR) of the identified pathways. The enriched pathways include other glycan degradation, motor proteins, carbon fixation by the Calvin cycle, pentose phosphate pathway, protein processing in the endoplasmic reticulum, glycolysis/gluconeogenesis, spliceosome, fructose and mannose metabolism, endocytosis, biosynthesis of amino acids, carbon metabolism, biosynthesis of secondary metabolites, and general metabolic pathways. These results indicate that tomato-derived EVs are associated with diverse biological processes, including carbohydrate metabolism, protein processing, vesicle trafficking, and cellular transport mechanisms. Notably, the enrichment of pathways such as endocytosis and protein processing suggests a role for EVs in intracellular communication and molecular transport, while metabolic pathways reflect their involvement in cellular homeostasis and stress adaptation. Collectively, these findings support the functional relevance of EV cargo in coordinating metabolic and regulatory processes within plant cells**.**

### Protein–protein interaction via STRING database

**Fig. 8:**
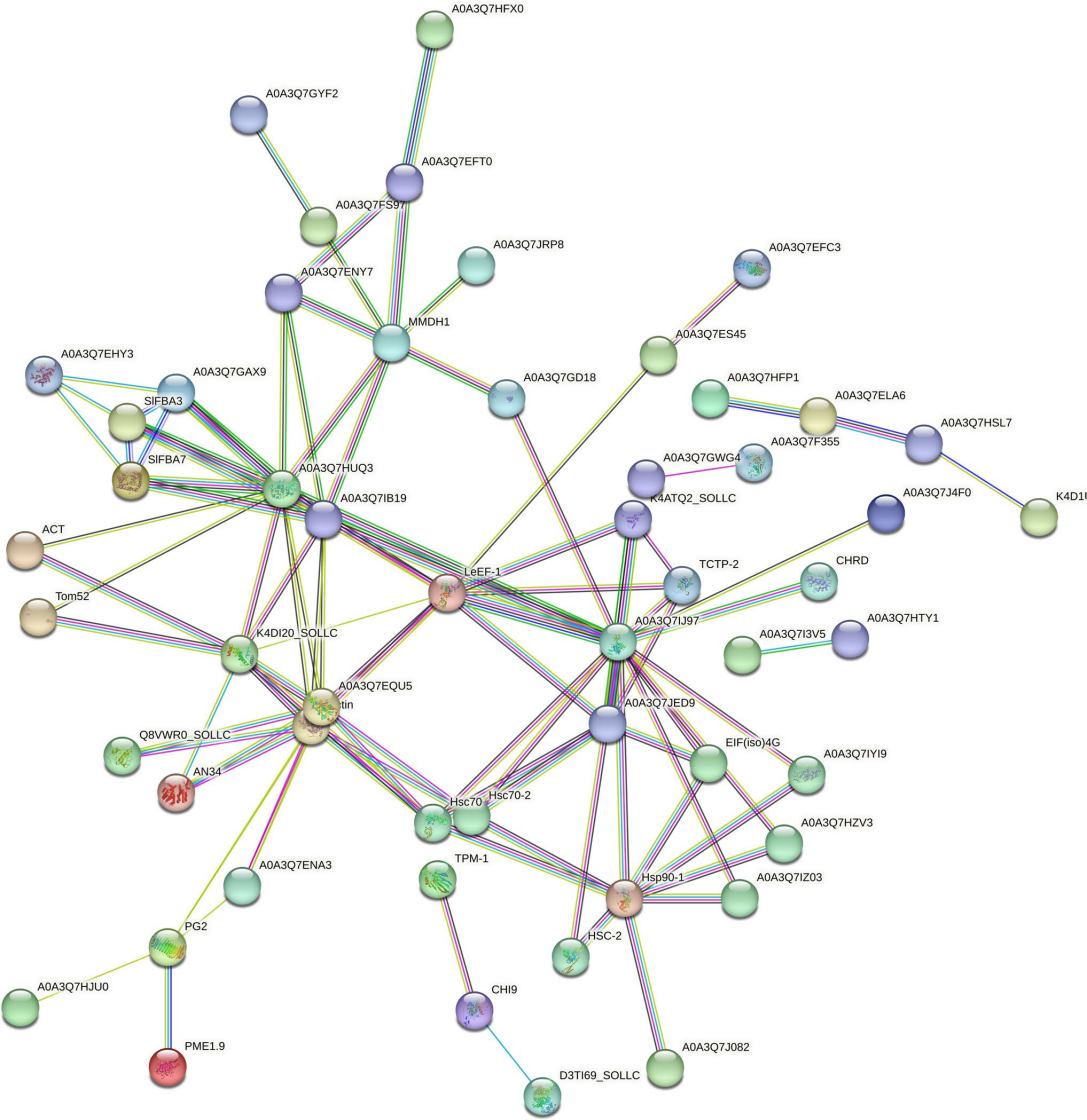
Protein–protein interaction network of *Solanum lycopersicum*-derived exosomal proteins generated using STRING database (version 11.5; https://string-db.org), showing significantly enriched interactions among identified proteins. Sphere colors represent functional biological clusters determined by network analysis (e.g., green spheres represent cell wall modification proteins, blue spheres represent protein synthesis and translation machinery, and red/pink spheres represent stress response and chaperone proteins). Key hub proteins are labeled with their standard abbreviations or UniProt accessions, including LeEF-1 (elongation factor 1-alpha), HSC-2 (heat shock cognate 70 kDa protein 2), and A0A3Q7EFC3 (importin subunit alpha). Full names, descriptions, and accession numbers for all visualized proteins are detailed in Supplementary File S1

We conducted protein–protein interaction (PPI) analysis of the identified protein utilizing the STRING database. The resultant network (Fig. 8) exhibited a markedly greater number of interactions than anticipated for a randomly selected set of equivalent size from the genome, indicating a functional intersection of the EV proteins (expected number of edges: 36; actual number of edges: 101; enrichment *P*-value < 1.0e-16; confidence score: moderate, 0.4).

### Antifungal activity of *S. lycopersicum* exosomes

In the disk diffusion assay, no visible inhibition zones were observed for tomato-derived EVs against *Fusarium oxysporum*, *Fusarium solani*, or *Botrytis cinerea*. This observation may be attributed to the limited diffusion capacity of EVs in solid agar media, which can restrict the interaction between vesicle-associated bioactive molecules and fungal cells. Consequently, antifungal activity was further evaluated using liquid-based assays.

### Effect of *S. lycopersicum* exosomes on fungal spore density and germination

**Fig. 9:**
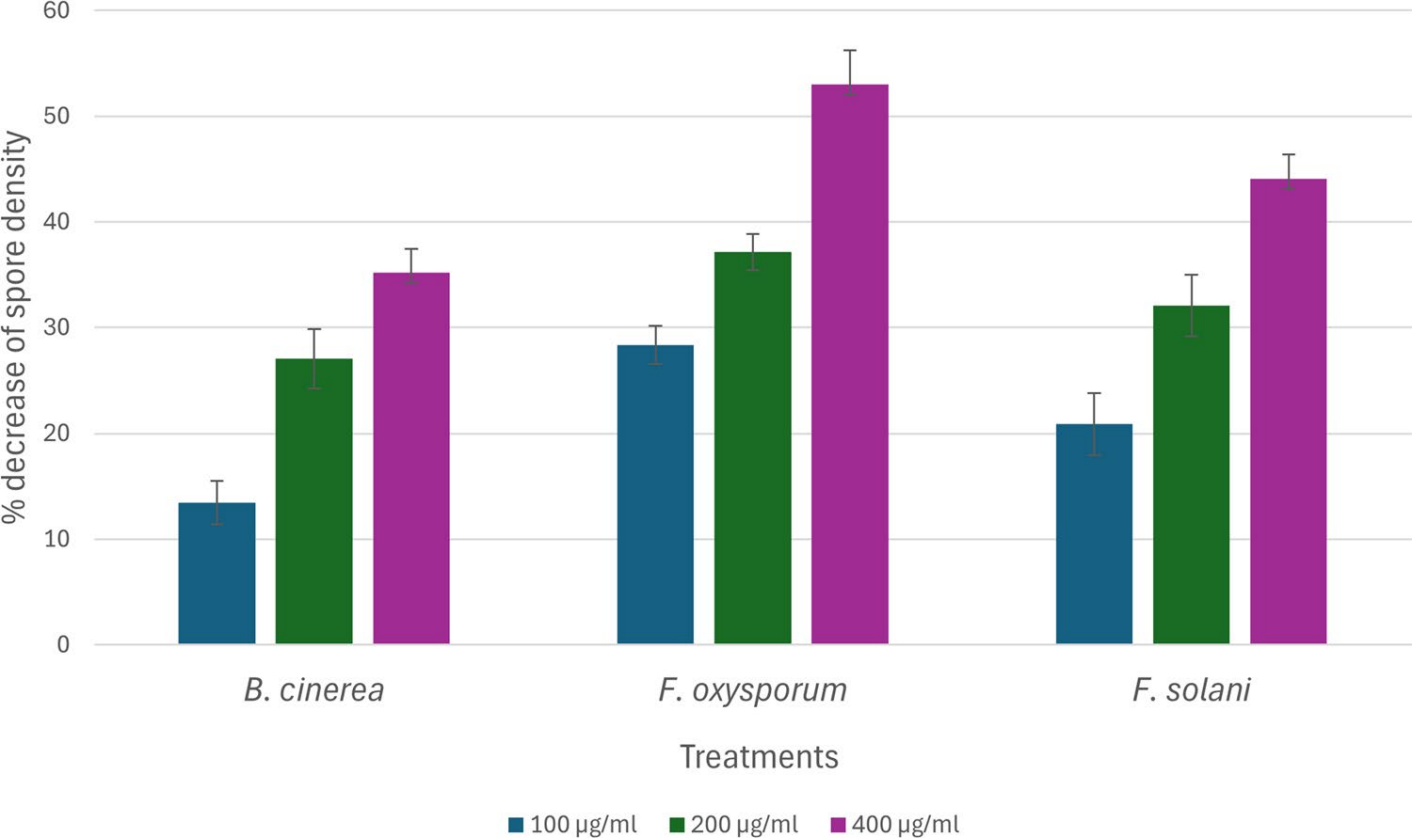
Effect of tomato-derived extracellular vesicles on fungal spore density. Data are expressed as a percentage (%) reduction in spore density relative to the untreated control. Values represent mean ± standard error (SE) of three independent experiments

**Fig. 10:**
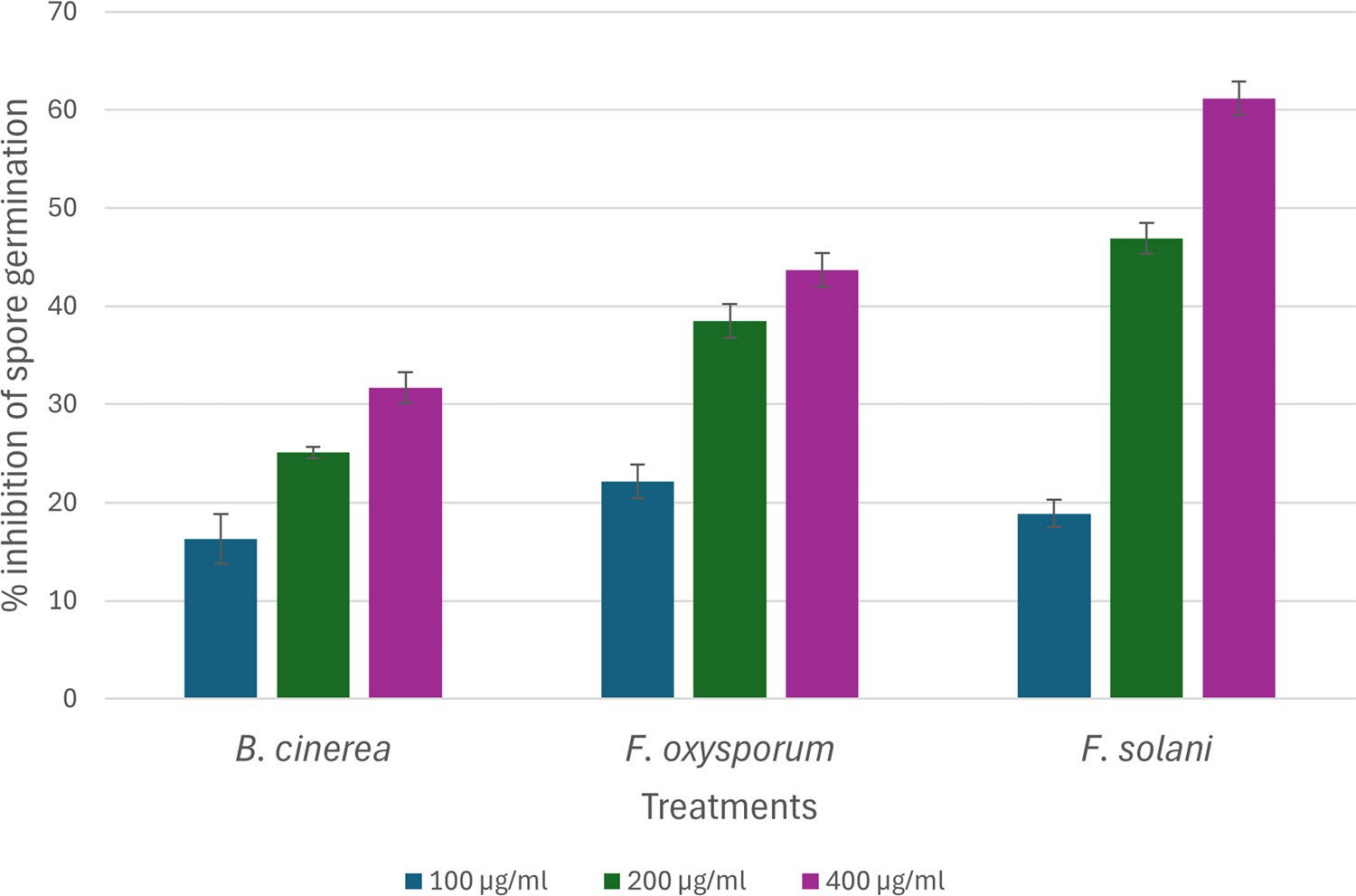
Effect of tomato-derived extracellular vesicles on fungal spore germination. Data are expressed as a percentage (%) reduction in spore germination relative to the untreated control. Values represent mean ± standard error (SE) of three independent experiments

The antifungal activity assessment revealed that tomato-derived exosomes significantly reduced the density and germination of *F. oxysporum, F. solani*, and *B. cinerea* spores (Figs. 9, 10). The most significant reduction in spore density occurred when *F. solani* spores were treated with exosomes generated from *S. lycopersicum* at 400 µg/ml*,* resulting in a 71.33% reduction compared to the control. Moreover, the greatest reduction in spore germination was observed when spore solutions of the same species were treated with 400 µg/ml of *S. lycopersicum-derived* exosomes, resulting in a 61.16% decrease compared to the control.

## Discussion

The purpose of this research was to determine whether EVs derived from tomatoes possess a distinct antifungal proteome signature and whether this payload inhibits fungal development. We aimed to determine whether EVs function as organized antifungal delivery systems rather than merely passive extracellular secretions by integrating proteome profiling with antifungal assays.

Characterization of the isolated vesicles confirmed the presence of nanoscale membrane-bound particles consistent with previously described plant EVs. Transmission electron microscopy revealed vesicles with diameters ranging from 95 to 231 nm, which fall within the size range reported for plant EVs. Dynamic light scattering analysis detected two particle populations. This apparent discrepancy between TEM and DLS measurements is commonly observed because DLS measures hydrodynamic diameter in suspension and produces intensity-weighted distributions that are highly sensitive to larger particles or minor aggregation. Similar heterogeneous size distributions have been reported for EVs isolated from several plant species, highlighting the intrinsic heterogeneity of EV populations (Samaeekia et al. 2018; Ma et al. 2024; Naselli et al. 2024). Compared to animal-derived exosomes, plant-derived exosomes have a relatively wider particle size distribution, with an average particle diameter ranging from 30 to 400 nm (Lee et al. 2020).

Proteomic analysis of tomato fruit exosomes identified 133 proteins involved in various biological processes, including stress response, carbohydrate metabolism, vesicular trafficking, and protein folding. Among these identified proteins, many of the exosome markers, including heat shock protein 90, and 70, actin, enolase, 14–3-3 protein 4, Fas ligand (FasL), SNAREs, syntaxin-related protein knolle, ubiquitin, annexin, glyceraldehyde-3-phosphate dehydrogenase, pyruvate, phosphate dikinase (DeCastro et al. 2021). Proteomic analysis revealed 133 high-confidence proteins implicated in several biological processes, such as protein homeostasis, stress response mechanisms, cellular structural organization, and supporting metabolic regulation. The identification of conventional EV-associated proteins, including heat shock proteins, annexins, actin, and small GTPases, substantiates the effective enrichment of vesicular components over nonspecific extracellular pollutants. These proteins are recognized for their roles in vesicle formation, cargo sorting, and membrane trafficking mechanisms associated with MVBs and the endomembrane system (Rutter And Innes 2018).

Multiple proteins previously associated with antifungal defense were identified within purified EVs, as illustrated in Table 1, which is particularly pertinent to the central research question. These were PR proteins, defensin proteins, endochitinase, glucanase-related enzymes, osmotin-like proteins (PR-5 family), lipid transfer proteins, xyloglucan-specific-fungal endoglucanase inhibitor protein. Chitinases and glucanases decompose structural elements of fungal cell walls (Boudart et al. 2005), defensins compromise fungal membranes, and lipid transfer proteins can affect pathogen membrane integrity and signaling mechanisms (Pusztahelyi 2018; Gao et al. 2022a, b). The presence of these defensive proteins in vesicle cargo suggests that EVs may deliver a coordinated array of antifungal proteins capable of simultaneously targeting multiple fungal cellular processes.

Despite the extensive characterization of many proteins in detailed secretome or apoplastic fluid investigations, their identification in pure EV fractions supports the concept that antifungal proteins are selectively contained within vesicles. This distinction is paramount. Conventional extracellular protein analysis cannot distinguish between freely released proteins and vesicle-encapsulated cargo. In the absence of proteome profiling specific to EVs, defense proteins abundant in vesicles and their coordinated delivery methods may remain undetected. Our findings indicate that tomato EVs are not only cellular waste but structured carriers of defense-related proteins.

The existence of endochitinase, glucanases, and defensins indicates multiple mechanisms to combat fungi. (1) Cell wall degradation: chitinases and glucanases can decompose components of the fungal cell wall (Grover 2012). (2) Membrane disruption: defensins and lipid transport proteins can alter membrane permeability (McLaughlin And Tumer 2025). (3) Enzyme inhibition: Proteins that inhibit xyloglucan-specific endoglucanase may impede enzymes responsible for degrading fungal cell walls (Xie et al. 2008; Jian et al. 2024; Marcianò et al. 2025). (4) Stress modulation: osmotin-like proteins and PR proteins may interfere with fungal osmotic control (Sripriya et al. 2017; dos Santos And Franco 2023; Han And Schneiter 2024). The coexistence of several proteins within EV cargo indicates a synergistic antifungal approach that addresses multiple targets, rather than the secretion of a singular protein.

KEGG enrichment and gene ontology analysis indicated that pathways related to: carbohydrate metabolism, protein processing, vesicle trafficking, and cellular transport mechanisms. The enrichment of pathways such as endocytosis and protein processing indicates that EVs participate in intracellular communication and molecular trafficking, whereas the prominence of metabolic pathways highlights their role in maintaining cellular homeostasis and facilitating adaptive responses to stress. Using the entire *Solanum lycopersicum* proteome as a reference, we demonstrate that defense-related pathways are markedly enriched relative to the overall protein population. This enrichment facilitates selective cargo packaging instead of random extracellular protein release.

The identification of vesicular transport proteins (annexins, small GTPases, ESCRT-related proteins) suggests that tomato EVs are actively generated through endomembrane trafficking pathways. This supports models indicating that, during stress, MVBs fuse with the plasma membrane to discharge their intraluminal vesicles into the apoplast (Yáñez-Mó et al. 2015; Li et al. 2025). This type of spatial arrangement may confer several advantages to plants: protecting labile proteins against extracellular breakdown, targeted delivery to specific infection areas, orchestrating the mobilization of many defensive chemicals, and potential assistance with RNA transfer across kingdoms. Thus, EV-mediated transport may represent an additional mechanism by which plants regulate their immune systems.

This research employed EVs derived from mature tomato fruits. This offers significant insight into the distinct payload of tissue-derived EVs; nonetheless, subsequent research should explore: proteomes of EVs from infected tissues, vesicle composition specific to developmental stages, quantitative analysis of EV cargo versus total secretome, functional analysis of individual antifungal proteins. Such experiments will elucidate whether selective packaging occurs dynamically following pathogen exposure.

EVs produced by tomatoes exhibited strong antifungal activity against *B. cinerea, F. oxysporum, and F. solani*, presumably due to the presence of defense-associated proteins, including PR proteins, defensins, glucanase inhibitors, osmotin-like proteins, and lipid transfer proteins. These results align with earlier research indicating antifungal protein enrichment in plant EVs (Rutter And Innes 2016) and their capacity to inhibit fungal proliferation and spore germination (Palma et al. 2020; Salem And Aziz 2025), along with comparable antifungal effects noted in EVs from other plant species.

The absence of inhibition zones in agar-based assays may reflect the limited diffusion of EVs in solid media, highlighting the importance of liquid-phase assays for evaluating vesicle-mediated bioactivity. Notwithstanding these findings, some limitations must be mentioned, including: the use of a single tomato cultivar, comparative analyses between resistant and susceptible genotypes are needed to better define the role of EV cargo in plant defense, in addition to lacking quantitative comparison with the complete proteome, which constrains conclusions on the selective enrichment of defense proteins in EVs. Although the presence of antifungal proteins and the observed suppression of fungal growth indicate a functional role for EVs, a direct molecular link has yet to be established. Future research utilizing quantitative proteomics methodologies (e.g., label-free quantification or isotopic labeling) is necessary to ascertain the extent of selective protein packaging into EVs. Tomato-derived EVs harbor defense-related proteins and may serve as organized carriers of antifungal compounds, providing a basis for forthcoming quantitative and mechanistic investigations of plant–pathogen interactions.

Despite these limitations, including the use of a single tomato cultivar and the absence of quantitative proteomic comparison, this study provides the first integrated analysis combining EV isolation, proteomic profiling, and antifungal functional assays in tomato fruit-derived vesicles. The observed inhibition of fungal spore germination is consistent with previous reports demonstrating antifungal activity of plant-derived EVs. Collectively, these findings support a potential role of EV-associated cargo in plant defense and provide a foundation for future comparative and mechanistic studies.

## Conclusion

This study demonstrates that tomato-derived EVs contain a proteomic repertoire enriched in defense-associated and antifungal-related proteins. By independently analyzing purified EV cargo, we provide evidence that vesicle-associated proteins contribute to suppression of fungal spore development and germination. These findings support the hypothesis that a potential role lies in coordinated defense rather than in passive extracellular remnants. Understanding the proteomic composition of plant EVs enhances our mechanistic insight into plant immunity and may inform the development of sustainable, vesicle-based biocontrol strategies.

## Supplementary Information

Below is the link to the electronic supplementary material.

Supplementary file1 (XLSX 3932 KB)

## Data Availability

Data are provided in supplementary information files.

## Abbreviations

DLS: Dynamic light scattering
EVs: Extracellular vesicles
FDR: False discovery rate
GO: Gene ontology
KEGG: Kyoto Encyclopedia of Genes and Genomes
MVBs: Multivesicular bodies
PR proteins: Pathogenesis-related proteins
TEM: Transmission electron microscopy
